# Incidental Prostate Cancer Is an Uncommon but Clinically Non-negligible Cause of Death in Selected Long-term Survivors of Urothelial Carcinoma—Oncological Outcomes and a Proposed Follow-up Strategy from a Tertiary Referral Center

**DOI:** 10.1016/j.euros.2026.02.004

**Published:** 2026-02-20

**Authors:** Marc A. Furrer, Benjamin Lyttwin, Michael S. Pärli, Fiona C. Burkhard, George N. Thalmann, Patrick Y. Wuethrich, Niall M. Corcoran

**Affiliations:** a Department of Urology, University of Bern, Bern, Switzerland; b Department of Urology, Solothurner Spitäler AG, Kantonsspital Olten, and Bürgerspital Solothurn, Solothurn, Switzerland; c Department of Anaesthesiology and Pain Medicine, Inselspital, Bern University Hospital, University of Bern, Bern, Switzerland; d Department of Urology, The University of Melbourne, The Royal Melbourne Hospital, Parkville, Victoria, Australia; e Department of Urology, Western Health, Melbourne, Victoria, Australia

**Keywords:** Incidental prostate cancer, Radical cystoprostatectomy, Urothelial carcinoma, Bladder cancer, Long-term survival, Prostate-specific antigen surveillance, Tumor recurrence

## Abstract

**Background and objective:**

Incidental prostate cancer is frequently identified in men undergoing radical cystoprostatectomy for urothelial carcinoma of the bladder. While usually clinically insignificant, its long natural history raises questions about long-term impact as systemic therapies prolong bladder cancer survival. The aim of this study is to describe the natural history, recurrence patterns, and survival outcomes of prostate cancer detected incidentally following radical cystoprostatectomy in a prospective cohort, and to provide a follow-up protocol for these patients.

**Methods:**

A retrospective cohort study with prospective follow-up of consecutive men undergoing radical cystoprostatectomy with urinary diversion for urothelial carcinoma at a tertiary referral center (1999–2020) was conducted. Follow-up included serial prostate-specific antigen (PSA) testing, imaging, and cause-specific survival. Associations with recurrence and survival were assessed using Cox regression.

**Key findings and limitations:**

Incidental prostate cancer was diagnosed in 384 of 940 men (41%). Prostate cancer recurrence was reported in 23 men (6%) at a median of 25 mo, most often in bone and lymph nodes. Seven patients died of prostate cancer at a median of 72 mo after surgery. Risk factors for recurrence included higher International Society of Urological Pathology grade (hazard ratio [HR] 2.9, 95% confidence interval [CI] 2.2–3.9; *p* < 0.001), locally advanced stage (HR 6.8, 95% CI 4.1–11.2; *p* < 0.001), preoperative PSA (HR 2.3, 95% CI 1.4–3.8; *p* = 0.002), and nodal metastasis (HR 29.3, 95% CI 10.8–79.3; *p* < 0.001), although HR effect estimates were exploratory and based on a limited number of events. Overall survival was determined by bladder cancer stage, comorbidity, and age; prostate cancer features were prognostic only beyond 3 yr.

**Conclusions and clinical implications:**

Incidental prostate cancer after radical cystoprostatectomy is usually organ confined and of low grade, with a low short-term risk. In long-term survivors with adverse pathological features, incidental prostate cancer represents a clinically non-negligible contributor to cause-specific mortality, despite a low absolute risk at the population level. Selective risk-adapted PSA surveillance should be considered in patients with higher-risk pathology and favorable bladder cancer outcomes following a protocol.

**Patient summary:**

We studied men with prostate cancer diagnosed incidentally during bladder cancer surgery. While most cases of prostate cancer were at a low risk and rarely caused early problems, some patients with higher-risk prostate cancer developed metastases or died from the disease later. Careful follow-up for prostate cancer may benefit selected long-term bladder cancer survivors.

## Take Home Message

Incidental prostate cancer is detected frequently in men undergoing radical cystoprostatectomy for bladder cancer and is usually of low risk. While short-term outcomes are dominated by urothelial carcinoma, advanced prostate cancer features contribute to late mortality. Selective prostate-specific antigen surveillance should be considered in long-term survivors with higher-risk prostate cancer pathology.

## 1 Introduction

Incidental prostate cancer (iPCa) is a common finding in men undergoing radical cystoprostatectomy for urothelial carcinoma of the bladder, with a meta-analysis of over 13 000 patients reporting a prevalence rate of 24.4% (95% confidence interval [CI] 22.4–34.4) [1]. Patients diagnosed with prostate cancer were found to be significantly older at the time of surgery, and in two-thirds of patients prostate cancer was judged to be clinically insignificant, with low rates of disease recurrence and progression. Overall survival was significantly lower in men with iPCa than in those without, although in the absence of granular follow-up and survival data, this likely reflects higher competing mortality risks in a more elderly population. These findings are largely supported by more recently published retrospective single-center [2], international multicenter [3], and population-based [4] series, leading to the suggestion that prostate cancer surveillance can largely be omitted during follow-up—if not universally, then at least for the vast majority of patients.

The treatment landscape of muscle-invasive bladder cancer, however, continues to evolve, with advances in systemic management leading to improvements to overall survival. Against this backdrop, further consideration needs to be given to the impact of a disease like prostate cancer, which has a long natural history, has on overall survival. A common limitation of the studies performed to date is the lack of a standardized regular follow-up protocol and/or incompleteness of data for critical endpoints. For instance, in the meta-analysis, important clinical features such as bladder tumor grade was available for just over a third of patients, and only a slightly higher number of patients were actually followed for prostate cancer recurrence [1]. Similarly, in the series reported by Packiam et al [2], only 60% of patients underwent prostate cancer surveillance postoperatively.

We were therefore motivated to describe the natural history of prostate cancer detected incidentally in men undergoing radical cystoprostatectomy for urothelial carcinoma of the bladder within a prospective observational study with systematic data collection. Based on these findings, we aim to develop a structured follow-up protocol that can be individualized according to the patient’s specific prostate and bladder cancer risk profile.

## 2 Patients and methods

This retrospective cohort study with prospective follow-up included consecutive men who underwent radical cystoprostatectomy with urinary diversion between January 1999 and July 2020 at the Department of Urology, Inselspital Bern, a tertiary high-volume reference center for bladder cancer. Clinical, pathological, and follow-up data were recorded longitudinally in a structured institutional registry, while patient identification and the present analysis were performed retrospectively.

Incidental prostate cancer was identified in the cystoprostatectomy specimen. Patients with a known diagnosis of prostate cancer prior to cystoprostatectomy—either on active surveillance or after definitive treatment—were excluded by definition, as the study focused exclusively on prostate cancer diagnosed incidentally. The study followed the STROBE statement and was approved by the Ethics Committee of the Canton Bern (KEK-Be 2016-00660).

### 2.1 Data collection

Data were collected as per the Declaration of Helsinki, Good Clinical Practice, and local regulations. Follow-up information was derived from a prospectively maintained clinical registry in the form of a structured electronic database; although completeness varied across variables, particularly for preoperative assessments performed outside the standardized protocols, completeness for pathological and long-term follow-up data that have been in continuous use for routine patient care and quality assurance is ensured. For iPCa cases, aggregate data on recurrence sites, survival metrics, and cause-specific mortality were compiled.

### 2.2 Preoperative staging

Preoperative assessment included physical examination, prostate-specific antigen (PSA) measurement, and standard staging with computed tomography (CT)/magnetic resonance imaging (MRI) of the abdomen/pelvis, chest CT, bone scintigraphy, and examination under anesthesia [5]. Additional targeted imaging was performed if indicated. Suspected metastases upon imaging were biopsied (if technically feasible) if these remained unclear after subsequent targeted imaging to confirm metastatic disease [6].

### 2.3 PSA testing and preoperative prostate assessment

Preoperative PSA testing was not standardized during the early study period and was performed at the discretion of the treating physician, resulting in incomplete availability of PSA data. Preoperative assessment underwent further diagnostic workup, including prostate biopsy, at the discretion of the treating physician. By definition, patients with a known diagnosis of prostate cancer were excluded from this study.

### 2.4 Surgical technique and perioperative management

Radical cystoprostatectomy was performed according to institutional standards [7]. Nerve sparing was excluded in cases of palpable induration, intraoperative evidence of infiltration, or fibrosis. When feasible, unilateral or bilateral nerve sparing was performed depending on tumor location and stage [7].

### 2.5 Pathological assessment

Pathological examination of cystoprostatectomy specimens followed the established International Society of Urological Pathology (ISUP)/World Health Organization standards and mirrored the principles applied to radical prostatectomy specimens. The prostate was serially sectioned in its entirety, and iPCa foci were recorded systematically.

### 2.6 Postoperative follow-up

Patients were followed up at 3, 6, 12, 18, 24, 30, and 36 mo, and yearly thereafter. Routine follow-up included PSA, alkaline phosphatase, and liver function assessments, and imaging when indicated. Bone scintigraphy and CT were performed at 6, 12, and 24 mo in ≥T3 and/or N+ iPCa patients [6].

### 2.7 Follow-up for iPCa

All patients with prostate cancer diagnosed incidentally underwent postoperative PSA testing at 3 and 12 mo after surgery, and annually thereafter for up to 5 yr, followed by PSA assessments at 7, 10, 15, and 20 yr. In case of a detectable PSA level at any time point, PSA testing was intensified according to PSA dynamics. For patients with organ-confined, node-negative iPCa, no routine imaging was performed in the absence of biochemical recurrence. Bone scintigraphy and CT were performed at 6, 12, and 24 mo in ≥T3 and/or N+ iPCa patients [6].

### 2.8 Biochemical and radiologically detectable recurrence

Biochemical recurrence was defined as a postoperative rise in PSA to >0.2 µg/l [8]. Radiologically detectable recurrence was defined as evidence of prostate cancer recurrence on imaging, including bone scintigraphy, computed tomography, magnetic resonance imaging, or prostate-specific membrane antigen (PSMA) positron emission tomography (PET)/CT, performed in the setting of biochemical recurrence or clinical suspicion.

### 2.9 Mortality data

Overall survival was defined as the time from surgery to death from any cause; cancer-specific survival was defined as the time till death from prostate cancer. Patients alive at the last follow-up were censored.

### 2.10 Statistical analysis

Continuous variables are reported as medians (interquartile range [IQR]) and categorical variables as frequencies. Associations between pathological features and outcomes were explored using univariable survival analyses, and results are reported with hazard ratios (HRs) and corresponding CIs to reflect statistical uncertainty. Kaplan-Meier curves were generated to visualize the time to event data for overall, bladder cancer–specific, and prostate cancer–specific mortality. Survival analyses used Cox regression, with censoring at the last follow-up if no event occurred or death from any cause for cancer-specific survival. A landmark analysis was performed on patients surviving 3 yr after cystectomy, with the cutoff chosen based on visual inspection of survival curves. All tests were two sided, with significance set at *p* < 0.05.

For a more detailed description of the patients and methods, see the Supplementary material.

## 3 Results

### 3.1 Patient cohort

From an initial cohort of 1557 patients (521 female) undergoing cystectomy, we identified 940 men who underwent radical cystoprostatectomy for primary urothelial cancers, of whom 384 (41%) were diagnosed with prostate cancer incidentally (Table 1). Almost every patient had high-grade pure urothelial cancer, with 253/384 (66%) having muscle-invasive disease at the final pathology. The median number of lymph nodes removed was 32 (IQR 22–43). Metastatic urothelial nodal disease was identified in 98/384 (26%). Of 384 patients, 57 (15%) received neoadjuvant chemotherapy, whereas 27 (7%) and two (0.5%) of them received adjuvant chemotherapy and immunotherapy, respectively.

**Table 1:** Patient characteristics

Characteristic		*n* = 384
Age	Median (IQR)	70 (65–76)
PSA (*n* = 221)	Median (IQR)	2.3 (1.1–4.8)
Charlson Comorbidity Index	Median (IQR)	2 (3–4)
ISUP GG, *n* (%)	1	270 (70)
	2	66 (17)
	3	21 (6)
	4	14 (4)
	5	13 (3)
PCa pT, *n* (%)	T2	347 (90.3)
	T3a	15 (4)
	T3b	20 (5.2)
	T4	2 (0.5)
PCa pN, *n* (%)	0	373 (97)
	1	11 (3)
Surgical margin PCa, *n* (%)	Negative	372 (97)
	Positive	12 (3)
Bladder cancer, *n* (%)	Urothelial carcinoma	342 (89)
	Mixed	31 (8)
	Other	11 (3)
BCa pT, *n* (%)	T0	51 (13)
	Ta	17 (4)
	T1	63 (16)
	T2	113 (30)
	T3a	37 (10)
	T3b	74 (19)
	T4	29 (8)
BCa pN, *n* (%)	0	286 (74)
	1	34 (9)
	2	42 (11)
	3	22 (6)
BCa grade, *n* (%)	Low	45 (12)
	High	339 (88)
CIS, *n* (%)	No	221 (58)
	Yes	163 (42)
BCa margin, *n* (%)	Negative	374 (97)
	Positive	10 (3)

BCa = bladder cancer; CIS = carcinoma in situ; GG = grade group; IQR = interquartile range; ISUP = International Society of Urological Pathology; PCa = prostate cancer; PSA = prostate-specific antigen.

Incidental prostate cancer was predominantly of low grade and organ confined. Most patients had ISUP grade group 1 disease (270/384, 70.3%) and pathological stage pT2 (347/384, 90.3%), and were node negative (373/384, 97.1%), while 37/384 (9.6%) had locally advanced disease and 11/384 (0.3%) had nodal metastatic disease at the final pathology. Preoperative PSA measurements were available for 221 patients, which was ≤4 ng/dl in 154 (68%) patients.

### 3.2 Patient outcomes

After a median (IQR) follow-up of 69 (20–133) mo, 278 (72%) patients had died and 106 (28%) remained alive. Urothelial cancer of the bladder was the cause of death in 124 patients (45%), with a median survival time (IQR) of 20 (10–37) mo. The next most common causes of death were cardiovascular events (*n* = 54, 19%), old age/general decline (*n* = 32, 11.5%), and respiratory disease (*n* = 11, 4%).

During postoperative follow-up, 31 patients developed biochemically recurrent prostate cancer, with 23 patients progressing to radiologically detectable prostate cancer at a median (IQR) time of 25 (11–111) mo. The most common sites of recurrence were bone (*n* = 15, 65%), lymph nodes (*n* = 13, 57%), and lung/liver/other regions (*n* = 10, 43%). Six of 23 (26%) patients had additional locally recurrent disease. Seventeen patients (21% [10/48] of ISUP 3–5 patients and only 2% [7/336] of ISUP <3 patients) were treated with first-line androgen deprivation therapy, most commonly goserelin alone (*n* = 12). No patients received any upfront combination with docetaxel or an androgen receptor pathway inhibitor. Four patients progressed to second-line hormonal therapy (enzalutamide, *n* = 2; abiraterone alone, *n* = 1; and abiraterone plus apalutamide, *n* = 1). Two patients received chemotherapy after androgen deprivation therapy, and no patient was treated with radioligand therapy. Seven patients died from prostate cancer at a median (IQR) of 72 (30–132) mo after surgery. Five of them had ISUP 4–5 prostate cancer, and one each had ISUP 2 and 3 disease. No patient with ISUP 1 disease died from prostate cancer.

### 3.3 Factors associated with prostate cancer recurrence/death

Factors associated with an increased risk of prostate disease recurrence on a univariable analysis included ISUP grade group (HR 2.9, 95% CI 2.2–3.9; *p* < 0.001), tumor stage (HR 6.8, 95% CI 4.1–11.2; *p* < 0.001), preoperative PSA level (HR 2.3, 95% CI 1.4–3.8; *p* = 0.002), and prostate cancer nodal metastasis at the time of cystoprostatectomy (HR 29.3, 95% CI 10.8–79.3; *p* < 0.001). Factors associated with an increased risk of prostate cancer–specific death included ISUP grade group (HR 4.2, 95% CI 2.3–7.6; *p* < 0.001), tumor stage (HR 73, 95% CI 8–631; *p* < 0.001), and prostate cancer nodal metastasis at the time of cystoprostatectomy (HR 143, 95% CI 15–1334; *p* < 0.001). There were too few clinical events to perform a robust multivariable analysis. Given the small number of prostate cancer–specific events, the resulting HR estimates are subject to considerable uncertainty and should be interpreted as exploratory signals rather than precise measures of effect size.

### 3.4 Factors associated with overall survival

**Fig. 1:**
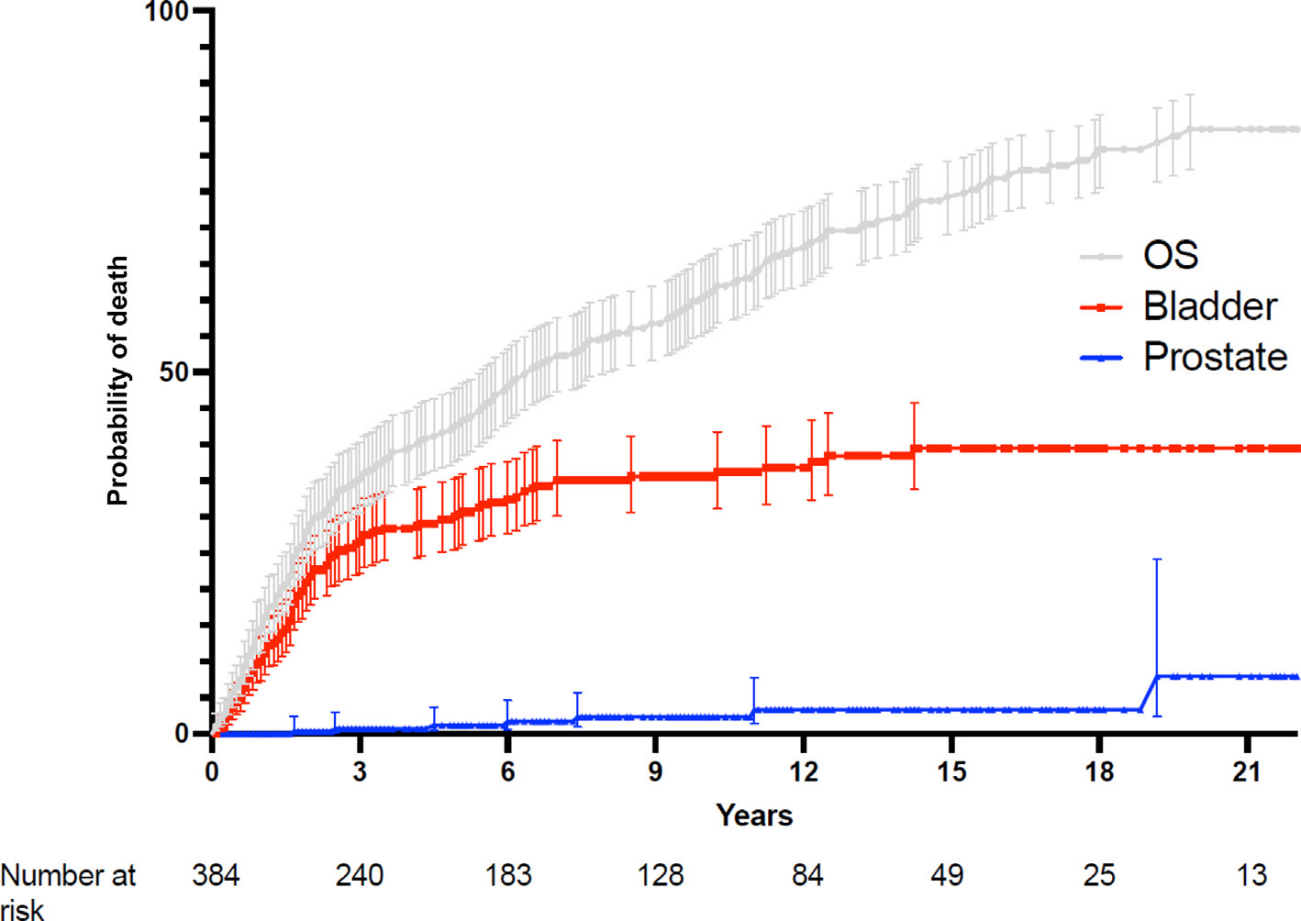
Overall survival and comparison of cancer-specific survival between patients with urothelial bladder cancer and incidental prostate cancer. The figure depicts patients with bladder cancer who experienced significantly worse CSS, with a 5-yr CSS rate of 73% compared with 99.3% in patients with incidental prostate cancer, and a 10-yr CSS of 69% versus 98.7%. Shaded areas represent 95% confidence intervals around survival estimates. CSS = cancer-specific survival; OS = overall survival.

The majority of deaths observed in the cohort occurred within the first 3 yr after surgery (60%, *n* = 141), of which 92 (65%) were from urothelial carcinoma and two (1.4%) from prostate cancer (Fig. 1). Factors significantly associated with overall survival are shown in Table 2 and include age, Charlson Comorbidity Index, and urothelial cancer stage and grade. Overall, no prostate cancer features were significantly associated with overall survival (Table 2). However, when the analysis was restricted to those patients who survived at least 36 mo following surgery, clinical features of prostate cancer began to impact survival significantly—in this case, organ-confined versus locally advanced disease (HR 2.95, 95% CI 1.3–6.6; *p* = 0.008; Table 3).

**Table 2:** Cox regression of overall survival

Characteristic	HR	95% CI	*p* value
Age	1.03	1.01–1.04	0.001
Charlson Comorbidity Index	1.17	1.08–1.27	<0.001
BCa tumor grade	0.92	0.62–1.35	0.657
BCa pT	1.81	1.39–2.37	<0.001
BCa pN	2.55	1.91–3.39	<0.001
ISUP GG	1.02	0.86–1.20	0.843
PCa pT	1.39	0.81–2.38	0.232
PCa pN	0.54	0.26–1.15	0.11

BCa = bladder cancer; CI = confidence interval; GG = grade group; HR = hazard ratio; ISUP = International Society of Urological Pathology; PCa = prostate cancer.

**Table 3:** Cox regression of overall survival for patients who survived at least 36 mo following surgery

Characteristic	HR	95% CI	*p* value
Age	1.05	1.03–1.08	<0.001
Charlson Comorbidity Index	1.22	1.08–1.39	0.002
BCa tumor grade	0.69	0.42–1.13	0.141
BCa pT	1.35	0.91–2.0	0.141
BCa pN	1.87	1.15–3.05	0.012
ISUP GG	1.00	0.79–1.27	0.975
PCa pT	2.95	1.33–6.57	0.008
PCa pN	0.65	0.24–1.81	0.411

BCa = bladder cancer; CI = confidence interval; GG = grade group; HR = hazard ratio; ISUP = International Society of Urological Pathology; PCa = prostate cancer.

## 4 Discussion

In this large prospective cohort study, we have confirmed that prostate cancer diagnosed incidentally in men undergoing cystoprostatectomy for urothelial carcinoma of the bladder is mainly organ confined and of low grade. More than two-thirds of patients harbored ISUP grade group 1 disease, over 90% had pathologically organ-confined tumors (pT2), and nodal involvement was uncommon, confirming observations from smaller institutional series and reinforcing the concept that iPCa detected in this setting is usually characterized by biologically favorable behavior [9]. In these patients, urothelial carcinoma is the most common cause of death in the short term, with clinical features recognized as risk factors for metastatic progression (extent of local invasion and nodal status) being significantly associated with overall survival after surgery. Deaths from urothelial carcinoma, however, start to decline after 3–5 yr before reaching a plateau, when other causes of death begin to dominate. Among these competing mortality risks is death from prostate cancer, which is relatively uncommon and tends to occur many years after surgery. At the same time, our data demonstrate that iPCa cannot be considered entirely irrelevant, particularly in patients who achieve long-term survival after bladder cancer treatment. Prostate cancer–specific mortality was rare, but when occurred, it was observed late during follow-up and predominantly in patients with adverse pathological features. This finding highlights the importance of extended follow-up when evaluating the clinical significance of iPCa, as prostate cancer is characterized by a long natural history that may only manifest clinically many years after cystoprostatectomy, often beyond the conventional surveillance period for bladder cancer.

Given the impact of bladder cancer–specific mortality on overall survival, improvements in the management of muscle-invasive bladder cancer that extend survival would be expected to increase the relative contribution of iPCa to overall deaths. For instance, in the VESPER study, patients with muscle-invasive bladder cancer treated with neoadjuvant dose-dense methotrexate, vinblastine, doxorubicin, and cisplatin achieved a 5-yr overall survival rate of 64%, compared with the rate of 56% in this series where the use of neoadjuvant and adjuvant therapy was low [10]. Similarly, in the recently published NIAGARA trial [11], the authors reported an overall survival rate of 82.2% at 24 mo in patients with muscle-invasive bladder cancer who received four cycles of durvalumab plus gemcitabine-cisplatin, followed by radical cystectomy and adjuvant durvalumab every 4 wk for eight cycles. This compares favorably with an overall mortality rate of 30% in our cohort at the same time point. It is likely that similar to metastatic melanoma [12] and lung cancer [13], many of these responses will be durable, increasing the proportion of men at risk of future demise from prostate cancer.

With this, the improving survival of patients with muscle-invasive bladder cancer further contextualizes our findings. Advances in perioperative and systemic therapy, including the introduction of antibody-drug conjugates combined with immune checkpoint inhibitors, have improved outcomes in advanced urothelial carcinoma significantly (eg, enfortumab vedotin plus pembrolizumab) [14], [15]. As bladder cancer–specific mortality decreases, competing causes of death become increasingly relevant, and prostate cancer—despite its generally indolent nature with a low absolute number of prostate cancer–related events—may emerge as a clinically meaningful issue in selected long-term survivors. Keeping this in mind, PSA-based follow-up is feasible and minimally burdensome, and can be incorporated into routine postcystectomy care. Although definitive evidence that earlier detection improves outcomes is lacking, a risk-adapted surveillance approach appears justified to identify the small subset of patients who may develop clinically relevant disease, while avoiding unnecessary interventions in the majority.

The questions remain whether prostate cancer risk should be considered regularly during preoperative evaluation and surgical planning in patients undergoing radical cystoprostatectomy, particularly when an orthotopic neobladder is contemplated. Suspected or clinically significant prostate cancer may limit apical and neurovascular preservation to ensure oncological safety, potentially compromising functional outcomes such as urinary continence. Therefore, patients with a higher prostate cancer risk should be counseled regarding the tradeoff between oncological control and functional expectations, and alternative urinary diversion options should be taken into account.

It is therefore reasonable to question whether men with muscle-invasive bladder cancer should be screened for localized prostate cancer prior to radical treatment. The most common method for screening is asymptomatic PSA testing; however, in our cohort, preoperative PSA was usually low and certainly below the traditional thresholds for a prostate biopsy. In addition, PSA may be falsely elevated due to instrumentation of the lower tract as well as previous intravesical therapy such as BCG instillation, which may also confound interpretation of MRI. In our survival analysis, the only prostate cancer clinical feature associated with overall survival was tumor stage (locally advanced vs organ confined), and it is possible that the knowledge of this would alter the extent of resection and influence the rate of positive surgical margins. In our analysis, although we found a positive association between prostate cancer tumor stage and a positive prostate cancer surgical margin, tumor at the margin was not associated with either prostate cancer recurrence or overall survival, with the caveat that the number of patients in this analysis is small. This would suggest that while the knowledge of a prostate cancer diagnosis and extent of disease preoperatively might be useful, the performance of screening tests may be affected adversely, and attempts to make a diagnosis should certainly not delay timely definitive treatment of their urothelial tumor.

A more pertinent question is the need for ongoing prostate cancer surveillance after radical cystoprostatectomy and lymph node dissection. The need for prostate cancer surveillance after radical cystoprostatectomy with lymph node dissection should be individualized and informed by both the pathological characteristics of the prostate cancer and the prognostic features of the concomitant urothelial carcinoma. Patients exhibiting adverse bladder cancer features—such as bulky residual disease or lymph node involvement—carry a high likelihood of mortality from urothelial carcinoma, rendering PSA surveillance largely redundant. Furthermore, ongoing prostate cancer surveillance should be abandoned whenever recurrence of urothelial carcinoma is documented.

Another key factor in selecting patients for surveillance following the diagnosis of iPCa is the management strategy for potential recurrence considering feasibility and benefit of treatment. Local recurrence is typically treated with external beam radiotherapy (EBRT). However, administration of EBRT after cystoprostatectomy—while technically feasible and a treatment option for recurrent urothelial carcinoma [16]—carries a high risk of complications due to altered pelvic anatomy, as reconstructed urinary diversions or small bowel loops often occupy the pelvic cavity and may be associated with cumulative pelvic radiation risk due to increased toxicity. Considering that survival remains favorable even in cases of local recurrence [17] and that symptoms are often absent due to urinary diversion, the therapeutic benefit of EBRT in this setting appears limited. On the contrary, emerging evidence suggests that local recurrence after definitive prostate cancer treatment often precedes metastatic progression and that outcomes after local salvage remain guarded [18]. In asymptomatic patients without radiographic evidence of metastatic disease, early initiation of systemic therapy may expose patients to treatment-related toxicity without a clear survival benefit. Therefore, systemic therapy would therefore be reserved for systemic progression at a low-volume stage or symptomatic disease in most cases when systemic therapy may be more effective and is normally well tolerated, rather than initiated solely on the basis of biochemical recurrence, further supporting a conservative surveillance approach.

An important clinical question is whether surveillance for iPCa is justified given the low absolute event rate. PSA testing is inexpensive, minimally invasive, and readily incorporated into routine postcystectomy follow-up, as patients are already monitored long term for bladder cancer recurrence and functional outcomes. As such, our data do not support intensive or universal surveillance, nor do these allow definitive conclusions regarding improved prostate cancer–specific survival through earlier detection of recurrence. Instead, a proportionate, risk-adapted surveillance strategy appears most appropriate, balancing the low overall risk against the potential burden of burdensome investigations. Given that the metastatic potential of organ-confined, low-grade prostate cancer (ISUP grade group 1) is effectively negligible [19], postoperative surveillance for prostate cancer recurrence is not warranted in these patients. Similarly, in most cases of ISUP grade group 2 disease, the likelihood of clinically significant recurrence remains exceedingly low, and when recurrence occurs, it typically manifests several years after initial therapy [20]. Therefore, routine surveillance may reasonably be omitted in this group as well. Nonetheless, certain pathological parameters may confer an increased individual risk of recurrence, including positive surgical margins, high total tumor volume, a greater proportion of Gleason pattern 4, the presence of adverse architectural subtypes such as large cribriform or intraductal carcinoma, and additional unfavorable histopathological findings such as lymphovascular invasion. For such patients, a tailored surveillance strategy initiated 5 yr after cystoprostatectomy may be considered, particularly when life expectancy exceeds 10 yr.

This leaves a small subgroup of patients with favorable bladder cancer prognosis but adverse prostate cancer features—namely, ISUP ≥3 (grade groups 3–5) and/or pathological stage ≥T3, and/or patients with prostate cancer lymph node metastasis—in whom PSA surveillance is clearly warranted.

Two strategies are proposed: first, a standard protocol—baseline PSA at 3 mo, repeat at 12 mo, and then annually lifelong, and second, a more conservative protocol—PSA testing deferred until 5 yr and then annually lifelong, appropriate for older or comorbid patients. Since the aim of surveillance is early identification of low-volume metastatic disease, once biochemical recurrence (PSA ≥0.2 ng/ml) is documented, there is no need for immediate imaging, but intensification of PSA testing every 3–6 mo is recommended with regular calculation of PSA doubling time to distinguish systemic from local recurrence. As such, there is a potential role of PSA doubling time as an adjunct for earlier imaging and escalation of surveillance. In line with the current European therapeutic guidelines [21] and pivotal landmark trials in metastatic prostate cancer [22] as well as nonmetastatic castration-resistant prostate cancer [23], and with the aim of avoiding premature imaging and initiation of systemic therapy based solely on biochemical recurrence rather than true systemic progression, a conservative surveillance strategy is supported. Accordingly, PSMA PET/CT should be deferred until the PSA doubling time decreases to below 6–9 mo.

**Fig. 2:**
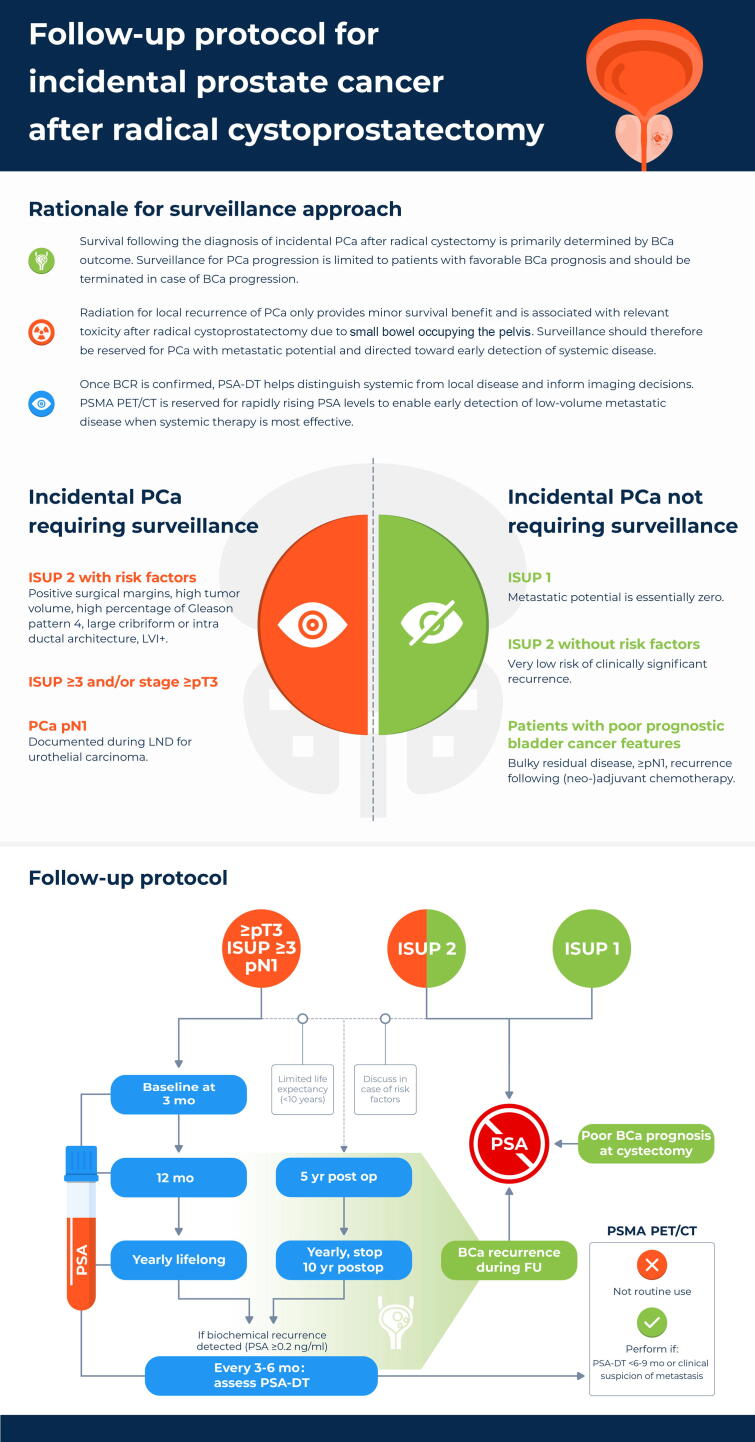
Follow-up protocol for incidental prostate cancer after radical cystoprostatectomy. BCa = bladder cancer; BCR = biochemical recurrence; FU = follow-up; ISUP = International Society of Urological Pathology; LND = lymph node dissection; LVI = lymphovascular invasion; PCa = prostate cancer; PSA-DT = prostate-specific antigen doubling time; PSMA PET/CT = prostate-specific membrane antigen positron emission tomography/computed tomography.

In summary, overall, lifelong surveillance is advised for ISUP ≥3 disease, while ISUP 2 disease, if monitored, may be discontinued at 10 yr or earlier if life expectancy is limited; however, patients with ISUP 1 disease and adverse bladder cancer prognosis or recurrence require no follow-up. A visual interpretation of this proposed follow-up routine is provided in Figure 2*.* As such, this study provides comprehensive, prospective characterization of iPCa in men undergoing radical cystoprostatectomy, highlighting the long-term risk of prostate cancer–specific mortality among bladder cancer survivors. Its findings can inform future research on individualized postoperative surveillance strategies, guide risk-adapted PSA monitoring protocols, and support investigations into the evolving interplay between improved bladder cancer survival and the clinical significance of iPCa.

To our knowledge, this study provides the most comprehensive prospective evaluation of iPCa in men undergoing radical cystoprostatectomy for bladder cancer. Leveraging a large, systematically collected cohort with standardized surgical and follow-up protocols—including PSA, imaging, recurrence, and survival data—our study overcomes the limitations of prior retrospective or incomplete datasets. It precisely characterizes the natural history of iPCa, showing that while most tumors are indolent, advanced pathological features significantly impact long-term prostate cancer–specific and overall mortality—insights largely unexplored in previous meta-analyses and multicenter series.

Several methodological limitations warrant acknowledgment. Although data were collected prospectively in a structured institutional registry using predefined electronic case report forms, the present analysis was retrospective in nature. Furthermore, PSA testing and preoperative prostate biopsy were not standardized and were performed at the discretion of the treating physician, which precludes definitive risk stratification. A selection bias is therefore possible and may have led to under-recognition of high-risk prostate cancer before surgery, and patients with known prostate cancer were excluded by definition. The absence of risk-adjusted or center-level analyses limits comparative interpretation, and center-specific variation could not be explored formally in the present study. These analyses are planned as dedicated follow-up studies and were intentionally beyond the scope of this overview. In addition, competing-risk methods were not used.

While pathological risk stratification, higher ISUP grade, extraprostatic extension, and nodal involvement were associated with an increased risk of recurrence and prostate cancer–specific death, the absolute number of prostate cancer–related events was small, limiting the statistical power of multivariable analyses for long-term outcomes. Consequently, the reported HRs—particularly the very large estimates observed for nodal disease—may be statistically unstable and reflect sparse-data effects rather than precise risk quantification. These findings should therefore be interpreted cautiously, with emphasis placed on the presence and direction of associations rather than the magnitude of individual HRs.

The long inclusion period of this cohort (1999–2020) is a central methodological consideration. During these 2 decades, major changes occurred in PSA testing patterns, imaging technologies, pathological grading systems, surgical techniques, and perioperative management of bladder cancer. Earlier patients were treated in an era of less frequent PSA testing, limited imaging sensitivity, and different Gleason grading criteria, whereas later patients benefited from multiparametric MRI, PSMA PET/CT, and refined pathological and staging standards. These temporal changes may have influenced the detection and timing of biochemical or radiographic recurrence, and the classification of metastatic disease. A further limitation is incomplete preoperative PSA availability (58%), reflecting historical practice variation. This may have led to under-recognition of high-risk prostate cancer and underestimation of clinically significant disease, warranting cautious interpretation of PSA-related analyses and surveillance recommendations.

However, the primary outcome of this study—prostate cancer–specific mortality—is a hard clinical endpoint that is less susceptible to a detection bias than biochemical recurrence or imaging-based progression. Although evolving diagnostic paradigms may have shifted the apparent timing of recurrence, these are unlikely to have substantially altered prostate cancer–specific death. In this regard, the long observational period represents not only a limitation, but also a strength, as it allows the assessment of clinically meaningful outcomes across eras and provides insight into the long-term natural history of iPCa in real-world practice. We also acknowledge that although the choice of 3 yr as the cut-point for the landmark analysis was based on the inspection of the bladder cancer survival curves, its selection remains arbitrary.

Taken together, the present study should be viewed as a landmark descriptive analysis rather than a definitive risk prediction model, and proposed surveillance algorithms involve a degree of clinical judgment and are not intended to function as rigid decision rules.

## 5 Conclusions

Prostate cancer is an uncommon but clinically non-negligible cause of death in selected long-term survivors of urothelial carcinoma among men diagnosed incidentally with the disease at the time of cystoprostatectomy. Routine postoperative risk-adapted surveillance for disease recurrence may be warranted in selected patients and should follow a protocol that is individualized according to both the patient’s specific prostate profile and the patient’s bladder cancer risk profile. Such an approach aligns with contemporary guideline principles that emphasize individualized follow-up, avoidance of overtreatment, and shared decision-making, particularly in patients with competing oncological risks.

  ***Author contributions*:** Marc A. Furrer had full access to all the data in the study and takes responsibility for the integrity of the data and the accuracy of the data analysis.

  *Study concept and design*: Furrer, Corcoran.

*Acquisition of data*: Furrer, Lyttwin, Pärli.

*Analysis and interpretation of data*: Furrer, Corcoran.

*Drafting of the manuscript*: Furrer, Lyttwin, Corcoran.

*Critical revision of the manuscript for important intellectual content*: All authors.

*Statistical analysis*: Corcoran.

*Obtaining funding*: None.

*Administrative, technical, or material support*: None.

*Supervision*: Corcoran.

*Other*: None.

  ***Financial disclosures:*** Marc A. Furrer certifies that all conflicts of interest, including specific financial interests and relationships and affiliations relevant to the subject matter or materials discussed in the manuscript (eg, employment/affiliation, grants or funding, consultancies, honoraria, stock ownership or options, expert testimony, royalties, or patents filed, received, or pending), are the following: None.

  ***Funding/Support and role of the sponsor:*** None.
