# Supplementary material for: Incidental Prostate Cancer Is an Uncommon but Clinically Non-negligible Cause of Death in Selected Long-term Survivors of Urothelial Carcinoma—Oncological Outcomes and a Proposed Follow-up Strategy from a Tertiary Referral Center
Source: Eur Urol Open Sci. 2026 Feb 20;86:10–9. doi: 10.1016/j.euros.2026.02.004 (PMC12937151; doi:10.1016/j.euros.2026.02.004)
Supplement: Supplementary Data 1 [file mmc1.docx]

**Supplementary File 1:**

**Patients and Methods**

This retrospective cohort study with prospective follow-up included consecutive men who underwent radical cystoprostatectomy with urinary diversion between 01/1999 and 07/2020 at the Department of Urology, Inselspital Bern, a tertiary high-volume reference center for bladder cancer. Clinical, pathological, and follow-up data were recorded longitudinally in a structured institutional registry, while patient identification and the present analysis were performed retrospectively.. Patients with a known diagnosis of prostate cancer prior to cystoprostatectomy—either on active surveillance or after definitive treatment—were excluded by definition, as the study focused exclusively on incidentally diagnosed prostate cancer. There was no preselection based on age, tumour stage, or surgical approach. The study was conducted in accordance with the “Strengthening the Reporting of Observational Studies in Epidemiology” (STROBE) statement and approved by the Ethics Committee of the Canton Bern, Switzerland (protocol number: KEK-Be 2016-00660) in June 2016, with amended approval in December 2018. The requirement for informed consent was waived. The study was officially registered at the University of Bern.

*Data collection*

Data were collected in accordance with the Declaration of Helsinki, Good Clinical Practice, local ethics and legal requirements. Follow-up information was derived from a prospectively maintained clinical registry in form of a structured electronic database resembling an electronic case report form, with predefined variables and explicit data-entry rules to ensure consistency of data capture, although completeness varied across variables, particularly for preoperative assessments performed outside standardized protocols while completeness for pathological, and long-term follow-up data that has been in continuous use for routine patient care and quality assurance is ensured. Follow-up data were additionally collected prospectively from general practitioners when patients were seen occasionally outside our institution. Follow-up data were additionally collected prospectively from general practitioners when patients were seen occasionally outside our institution. The dataset included among others:

- **Demographics:** age, sex, and year of surgery.
- **Oncological outcomes:** PSA values up to last follow-up, recurrence data (date and location of occurrence, and subsequent treatment [e.g., androgen deprivation treatment, radiation, chemotherapy, radionuclide / radioligand therapy ), survival time (in days, months, and years), date of last follow-up or death, and cause-specific mortality.
- **Postoperative imaging** (e.g., CT, MRI, bone scan, PSMA PET/CT)
- **Comorbidities:** Charlson Comorbidity Index (both age-adjusted and non–age-adjusted), presence of chronic conditions (e.g., diabetes, cardiovascular disease, COPD, AIDS, lymphoma).
- **Clinical history:** history of abdominal or genitourinary surgery, smoking status, and pack-years.
- **Tumor-related variables:** pathological staging, lymph node status, recurrence data, and iPCa findings.

Furthermore, we specifically compiled aggregate information on patients with iPCadetected at cystectomy, including number of cases, recurrence sites, survival metrics (means, medians, and confidence intervals), and cause-specific mortality.

*Preoperative staging*

Preoperative investigations included among others physical examination and measurement of PSA. Preoperative routine staging included CT and/or MRI of the abdomen/pelvis, CT scan of the chest, bone scintigraphy, and exam under anesthesia(1). Additional targeted imaging was performed if clinically indicated (e.g. if only a single positive or suspicious lesion was present in all staging exams in an otherwise locally confined disease) to evaluate for the presence of bone metastases. Suspected metastases upon imaging were biopsied (if technically feasible) if they remained unclear after subsequent targeted imaging to confirm metastatic disease(2).

*PSA testing and preoperative prostate assessment*

Preoperative PSA testing was not standardized during the early study period and was performed at the discretion of the treating physician, resulting in incomplete availability of PSA data. By definition, patients with a known diagnosis of prostate cancer were excluded from this study.

*Surgical technique and peri-operative management*

At our institution, open cystectomy and urinary diversion (ileal orthotopic bladder substitution, ileal conduit, catheterisable ileal reservoir, ureterocutaneostomy or ureterosigmoidostomy) have been performed following the same standardised surgical techniques for the last 10 years, as previously described(3).

Briefly, after cystectomy, using a standard intraperitoneal approach, a distal ileal segment was isolated and urinary diversion was performed with the ureters implanted into the proximal end of the ileal segment. The ureters were spatulated, implanted in an end to side fashion and splinted using mono-J ureteric stents(4). Pelvic lymph node dissection was carried out using an extended template, including the obturator, external iliac, internal iliac, and common iliac regions up to the ureteric crossing, in line with contemporary terminology and guideline recommendations.

Exclusion criteria of nerve sparing included palpable induration of the prostate (on digital rectal examination), intraoperative findings of adherence of the neurovascular bundle to the prostate (indicating tumor infiltration) and fibrosed neurovascular bundles. Whenever nerve sparing was attempted, it was performed on the non-bladder tumour-bearing side. Bilateral nerve sparing was offered for some unifocal tumours located in the ventral part of the bladder and in cases of non-muscle-invasive disease. In men, the dorsomedial bladder pedicle was transected along the ventral aspect of the seminal vesicles toward the base of the prostate, staying close to the vesicoprostatic angle and avoiding damage to the paraprostatic neurovascular bundle.

*Pathological assessment*

Pathological examination of cystoprostatectomy specimens followed established ISUP/WHO standards and mirrored the principles applied to radical prostatectomy specimens. The prostate was serially sectioned in its entirety, and incidental prostate cancer foci were recorded systematically. Tumours were graded according to the ISUP Grade Group system, and pathological staging was performed according to the TNM classification, including assessment of extraprostatic extension, seminal vesicle invasion, surgical margin status, and lymph node involvement.

*Postoperative follow-up*

Patients who underwent RC and urinary diversion had regular follow-up (clinical examination, blood analysis) according to our institutional protocol at 3, 6, 12, 18, 24, 30 and 36 months after surgery, and annually thereafter(3).

*Follow-up specifically for incidental prostate cancer*

All patients with incidentally diagnosed prostate cancer underwent postoperative PSA testing at 3 and 12 months after surgery, and annually thereafter up to 5 years, followed by PSA assessments at 7, 10, 15, and 20 years. In case of a detectable PSA level at any time point, PSA testing was intensified according to PSA dynamics. For patients with organ-confined, node-negative incidental prostate cancer, no routine imaging was performed in the absence of biochemical recurrence. Bone scintigraphy and CT were performed at 6, 12, and 24 months in ≥T3 and/or N+ incidental prostate cancer patients(6).

Restaging imaging was consistently performed in accordance with internationally accepted standards, with diagnostic modalities being continuously adapted to reflect scientific and guideline-based advances over time. Thus, while conventional cross-sectional imaging or choline-based PET/CT were applied in earlier years, more recently PSMA PET/CT has been implemented in patients with PSA levels > 0.2 µg/L(5).

*Biochemical and radiologically detectable recurrence*

Biochemical recurrence was defined as a postoperative rise in PSA to >0.2 µg/L(9). Radiologically detectable recurrence was defined as evidence of prostate cancer recurrence on imaging, including bone scintigraphy, computed tomography, magnetic resonance imaging, or PSMA PET/CT, performed in the setting of biochemical recurrence or clinical suspicion.

*Mortality data*

Overall survival was defined as the time from the date of cystoprostatectomy to death from any cause. Likewise, cancer-specific survival was defined as the time from the date of cystoprostatectomy to death from prostate cancer. Patients who were alive at the end of follow-up were censored at the date of their last recorded consultation.

*Statistical Analysis*

Continuous variables are reported as median and interquartile ranges. Categorical variables are reported as frequencies. Associations between pathological features and outcomes were explored using univariable survival analyses, and results are reported with hazard ratios and corresponding confidence intervals to reflect statistical uncertainty. Kaplan-Meier curves were generated to visualize time to event data for overall, bladder- and prostate-cancer specific mortality. Survival analyses used Cox regression, with censoring at last follow-up if no event occurred, or death from any cause for cancer-specific survival. A landmark analysis was performed on patients surviving 3 years post cystectomy, with the cut-off chosen based on visual inspection of survival curves. All tests were two-sided, with significance set at p<0.05.

**References**

1. Furrer MA, Papa N, Luetolf S, Roth B, Cumberbatch M, Dorin Vartolomei M, et al. A longitudinal study evaluating interim assessment of neoadjuvant chemotherapy for bladder cancer. BJU international. 2022;130(3):306-13.

2. Furrer MA, Grueter T, Bosshard P, Vartolomei MD, Kiss B, Thalmann GN, et al. Routine Preoperative Bone Scintigraphy Has Limited Impact on the Management of Patients with Invasive Bladder Cancer. European urology focus. 2021;7(5):1052-60.

3. Furrer MA, Roth B, Kiss B, Nguyen DP, Boxler S, Burkhard FC, et al. Patients with an Orthotopic Low Pressure Bladder Substitute Enjoy Long-Term Good Function. The Journal of urology. 2016;196(4):1172-80.

4. Furrer MA, Kiss B, Wüthrich PY, Thomas BC, Noser L, Studer UE, et al. Long-term Outcomes of Cystectomy and Crossfolded Ileal Reservoir Combined with an Afferent Tubular Segment for Heterotopic Continent Urinary Diversion: A Longitudinal Single-centre Study. European urology focus. 2021;7(3):629-37.

5. Furrer MA, Sathianathen NJ, Mulholland CJ, Papa N, Katsios A, Soliman C, et al. Pelvic Lymph Node Dissection in Prostate Cancer: Is It Really Necessary? A Multicentric Longitudinal Study Assessing Oncological Outcomes in Patients With Prostate Cancer Undergoing Pelvic Lymph Node Dissection vs Radical Prostatectomy Only. The Journal of urology. 2025;214(2):188-96.
